# A Systematic Analysis of the Role of Unc-5 Netrin Receptor A (UNC5A) in Human Cancers

**DOI:** 10.3390/biom12121826

**Published:** 2022-12-06

**Authors:** Zonglang Zhou, Bingfu Fan, Hongrong Cheng, Ming Wang, Jun Xie, Mingyuan Zou, Yi Yang

**Affiliations:** 1 Department of Internal Medicine, the Second Affiliated Hospital, Zhejiang University School of Medicine, Hangzhou 310000, China; 2 Department of Hepatobiliary and Pancreatic Surgery, Zhejiang Provincial People’s Hospital, Hangzhou 310000, China; 3 International Institutes of Medicine, the Fourth Affiliated Hospital, Zhejiang University School of Medicine, Yiwu 322000, China; 4 Department of Endocrinology, Yongding Hospital, Suzhou 215000, China; 5 Medical School, Southeast University, Nanjing 210009, China

**Keywords:** UNC5A, biomarker, tumor immunity, immunotherapy target

## Abstract

Unc-5 netrin receptor A (UNC5A), a netrin family receptor, plays a key role in neuronal development and subsequent differentiation. Recently, studies have found that UNC5A plays an important role in multiple cancers, such as bladder cancer, non-small cell lung carcinoma, and colon cancer but its pan-cancer function is largely unknown. Herein, the R software and multiple databases or online websites (The Cancer Genome Atlas (TCGA), The Genotype-Tissue Expression (GTEx), The Tumor Immune Estimation Resource (TIMER), The Gene Set Cancer Analysis (GSCA), Gene Expression Profiling Interactive Analysis (GEPIA), and cBioPortal etc.) were utilized to examine the role of UNC5A in pan-cancer. UNC5A was found to be highly expressed across multiple human cancer tissues and cells, was linked to clinical outcomes of patients, and was a potential pan-cancer biomarker. The mutational landscape of UNC5A exhibited that patients with UNC5A mutations had poorer progress free survival (PFS) in head and neck squamous cell carcinoma (HNSC) and prostate adenocarcinoma (PRAD). Furthermore, UNC5A expression was associated with tumor mutation burden (TMB), neoantigen, tumor microenvironment (TME), tumor microsatellite instability (MSI), immunomodulators, immune infiltration, DNA methylation, immune checkpoint (ICP) genes, and drug responses. Our results suggest the potential of UNC5A as a pan-cancer biomarker and an efficient immunotherapy target, which may also guide drug selection for some specific cancer types in clinical practice.

## 1. Introduction

Cancer poses a huge economic and disease burden worldwide due to its high morbidity and mortality rates [1,2]. Surgery, radiotherapy, chemotherapy, and immunotherapy (including Chimeric Antigen Receptor T-Cell Immunotherapy (CAR-T), adoptive cell therapy, and adoptive T cell therapy) are considered the four pillars of cancer treatment [3,4]. Despite some recent progress based on treatment strategies, the clinical outcomes of cancer patients remain poor. For example, as a hub cancer strategy, cancer immunotherapy enhances target recognition by the immune system and the elimination of cancer cells, thus achieving the purpose of cancer therapy [5,6,7,8]. However, only a few cancer types are suited for immunotherapy, such as lung cancer, liver cancer, and colorectal cancer, and the effectiveness and safety of cancer immunotherapy have limited its clinical application to a certain extent [9,10,11,12,13]. Therefore, assessing more specific and sensitive cancer prevention biomarkers and new therapeutic targets is necessitated.

Unc-5 netrin receptor A (UNC5A), encoding an axon-guiding molecule, is located on the 5q35 chromosomal region and functions as a receptor of netrin family (including netrin-1, and netrin-3) with key roles in neuronal development and differentiation [14,15]. Previous studies found that the UNC5A belong to dependence receptors, and its most notable functional feature is that UNC5A can act as a double-edged sword, its function depending on the availability of the ligands. When the ligand is absent, the receptor is in a monomeric state and the intracellular region is cleaved by caspases, leading to the release of the C-terminal portion, which initiates the downstream pathway that promotes apoptosis. In the presence of ligands, however, UNC5A form dimers that transmit various signals for cell survival, migration, and differentiation [16,17,18,19]. Recently, studies have found that UNC5A may function as a tumor suppressor, affecting the onset and progression of cancers. For instance, Silu Ding and colleagues found lowered UNC5A expression levels in non-small cell lung carcinoma compared to adjacent normal tissues. Moreover, they knocked down the UNC5A gene in human lung cancer cell lines and found enhanced cellular proliferation, invasion, migration, and apoptosis, while UNC5A overexpression derived opposite outcomes [14]. Another report illustrated that p53 can activate UNC5A expression by binding to the gene’s promoter, and overexpression of UNC5A greatly inhibits the colony formation ability of glioblastoma cells [20]. The tumor-suppressing role of UNC5A has also been confirmed in other cancer types, including bladder cancer and colon cancer [15,21]. However, only a few studies have illustrated its role in some specific cancer types, and the role of UNC5A in multiple human cancers remains unknown.

Herein, multiple online databases and R packages were used to evaluate the role of UNC5A in human cancers. We systematically illustrated the UNC5A mRNA expression profile, prognostic value, mutation patterns, gene enrichment of UNC5A and co-expressed genes, immune infiltration, relationships with various immunomodulators, tumor mutation burden (TMB), neoantigen, immune checkpoint (ICP) genes, tumor microenvironment (TME), and tumor microsatellite instability (MSI). Finally, we assessed the methylation status of UNC5A in pan-cancer and its correlations with drug sensitivity. Our findings indicated the potential of UNC5A as a prognostic biomarker and immunotherapy target, which may also influence drug sensitivity, thus, guiding the clinical use of drugs against tumors.

## 2. Methods

### 2.1. Expression Profile of UNC5A

The Tumor Immune Estimation Resource (TIMER) platform, a comprehensive tool for the systematic analysis of immune cell infiltrates in multiple cancer types, comprises different gene expression profiles between normal and tumor tissues from The Cancer Genome Atlas (TCGA) database [22,23]. Therefore, the TIMER online website was employed to evaluate the mRNA expression level of UNC5A in pan-cancer and normal tissues through the Wilcoxon test. Subsequently, clinical data and corresponding RNA sequences (including 33 types of cancer) were obtained from the TCGA database, and the mRNA expression of UNC5A between adjacent normal and cancer tissues was further analyzed in R software via Wilcoxon test and visualized through “ggplot2” package. The expression profiles of UNC5A mRNA in different cells (tumor and normal cells) were downloaded from the BioGPS database (http://biogps.org/#goto=welcome (accessed on 30 August 2022)). The results were visualized using Graphpad Prism (version 9.0.0 (SanDiego, CA, USA)). A *p*-value < 0.05 was considered statistically significant.

### 2.2. Prognostic Value of UNC5A in Pan-Cancer

The pan-cancer clinical information was collected from The Genotype-Tissue Expression (GTEx) and TCGA databases, and the relationships between UNC5A expression and progression-free interval (PFI), overall survival (OS), disease-free interval (DFI), and disease-specific survival (DSS), were assessed by Logrank test in R environment. Moreover, the prognosis predictive utility of UNC5A in pan-cancer was also analyzed in R environment through “timeROC” package (version 0.4). A *p*-value < 0.05 was considered statistically significant.

### 2.3. Genetic Alteration Analysis

The mutation status of UNC5A, as well as the progress free survival (PFS) across cancer types with or without UNC5A gene mutations, were determined on the cBioPortal database (https://www.cbioportal.org/ (accessed on 23 September 2022 )) via Logrank test [24,25]. After that, the mutation, copy number and expression of UNC5A in all samples were also analyzed by cBioPortal database via “OncoPrint” module. *p*-value < 0.05 indicated a statistically significant result.

### 2.4. UNC5A-Related Gene Enrichment Analysis

The STRING online platform (https://string-db.org/ (accessed on 1 October 2022)) was used to construct the protein-protein interaction (PPI) network for UNC5A and its related genes. Subsequently, the Kyoto encyclopedia of genes and genomes (KEGG) pathway enrichment and Gene Ontology (GO) enrichment analyses of UNC5A and related genes were conducted. First, we used the KEGG rest API (https://www.kegg.jp/kegg/rest/keggapi.html (accessed on 2 October 2022)) to obtain the latest gene annotations for the KEGG pathway. After that, the KEGG pathway was analyzed through R package “clusterProfiler” (version 3.14.3 (Guangchuang Yu, Guangzhou, China) [26]). As for GO, we used the GO annotation of genes from the R package “org.Hs.eg.db” (version 3.1.0 (Marc Carlson, Washington, DC, USA)); subsequently, the GO enrichment was also analyzed through R package “clusterProfiler”.

### 2.5. Relationships between UNC5A Expression and Immunomodulators, TMB, MSI, ICP Genes, TME, and Neoantigen

The correlations between UNC5A expression and different immunomodulators (chemokine, receptor, major histocompatibility complex (MHC), immunoinhibitor, and immunostimulators), ICP genes, TMB, MSI, neoantigen, and TME were evaluated online on SangerBox (http://sangerbox.com/home.html (accessed on 2 October 2022)) through Spearman correlation analysis. Differences with a *p*-value < 0.05 were considered statistically significant. In addition, in this study, correlations between variables were defined as follows: *r* < 0.3, weak correlation; *r* ≥ 0.3 and < 0.6, moderate correlation; and *r* ≥0.6, strong correlation.

### 2.6. Association of UNC5A Levels with Tumor Immune Infiltration Cells (TIICs) in Human Cancers

The associations of UNC5A levels with various TIICs were determined by performing Spearman correlation analysis using the “correlation” module of the TIMER online tool, with *p*-value < 0.05 indicating statistical significance.

### 2.7. UNC5A Methylation Profile and Its Correlation with Sensitivity toward Drugs

Over 750 small molecule drugs from Cancer Therapeutics Response Portal (CTRP) and Genomics of Drug Sensitivity in Cancer (GDSC), along with integrated 10,000 multi-dimensional genomic data across 33 cancer types from TCGA can be found on The Gene Set Cancer Analysis (GSCA) (http://bioinfo.life.hust.edu.cn/GSCA/#/ (accessed on 6 October 2022)) online website. First, the association of four methyltransferases, including DNMT3b, DNMT3a, DNMT2, and DNMT1, with UNC5A expression were analyzed through the Gene Expression Profiling Interactive Analysis online website (GEPIA, http://gepia.cancer-pku.cn/index.html (accessed on 4 October 2022)) via Pearson correlation analysis; after that the results were visualized in R software [27]. Subsequently, the association of the degree of methylation in UNC5A mRNA with its expression in pan-cancer was determined using the “Mutation” module in GSCA through Spearman correlation analysis. The correlation between the sensitivity of GDSC and CTRP drugs (top 30) and UNC5A expression in pan-cancer was assessed utilizing the “Drug” module in GSCA via Pearson correlation analysis. A false discovery rate (FDR) < 0.05 denoted statistical significance.

## 3. Results

### 3.1. UNC5A Is Highly Expressed in Pan-Cancer Tissues and Cell Lines

**Figure 1:**
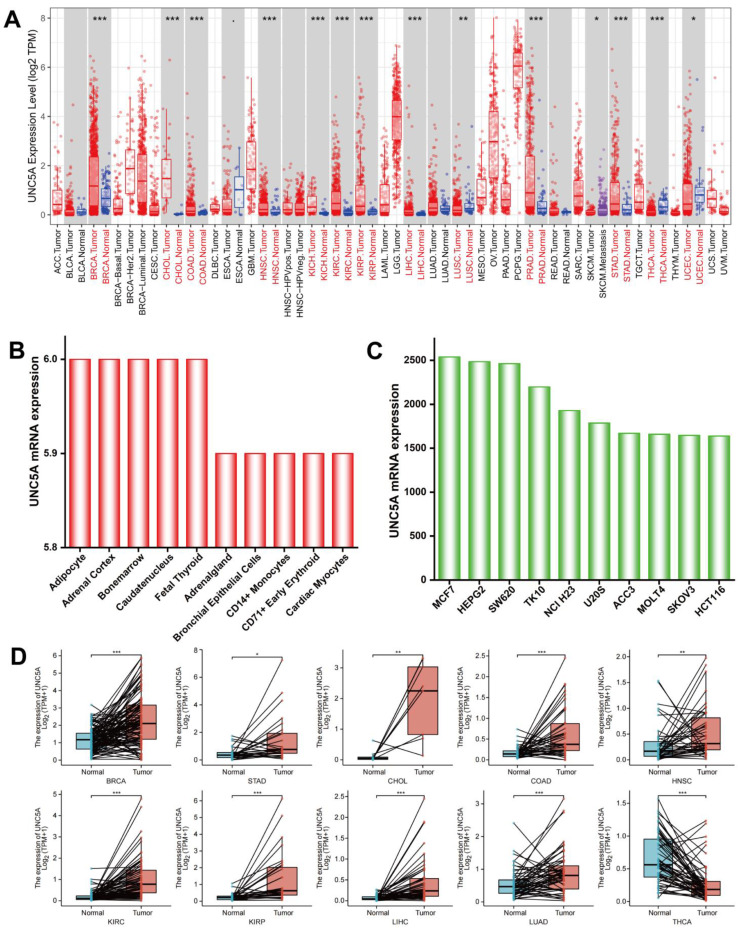
UNC5A mRNA expression profile between normal and cancer tissues and cells. (**A**) UNC5A mRNA expression profile between pan-cancer and normal tissues in TIMER online website. (**B**,**C**) UNC5A mRNA expression profile between normal and cancer cells (TOP 10). (**D**) UNC5A mRNA expression profile between pan-cancer and adjacent normal tissues in TCGA. * p < 0.05, ** p < 0.01, *** p <0.001.

UNC5A mRNA was highly expressed in colon adenocarcinoma (COAD), head and neck squamous cell carcinoma (HNSC), liver hepatocellular carcinoma (LIHC), kidney renal papillary cell carcinoma (KIRP), cholangiocarcinoma (CHOL), stomach adenocarcinoma (STAD), kidney renal clear cell carcinoma (KIRC), breast invasive carcinoma (BRCA), kidney chromophobe (KICH), and prostate adenocarcinoma (PRAD), whereas its expression was low in uterine corpus endometrial carcinoma (UCEC), thyroid carcinoma (THCA), and lung squamous cell carcinoma (LUSC) (Figure 1A), according to the TIMER data. Subsequently, we downloaded the RNA sequences from the TCGA database to further verify the expression pattern of UNC5A between adjacent normal and pan-cancer tissues. UNC5A was highly expressed in STAD, BRCA, COAD, CHOL, KIRC, HNSC, KIRP, and LIHC, while its expression was low in THCA. High expression in lung adenocarcinoma (LUAD) was found in the TCGA database (Figure 1D). The UNC5A mRNA expression profile between normal and cancer cells was assessed, and the results exhibited higher levels in cancer cells than in normal cells. The top ten normal and cancer cells with the highest expression of UNC5A are illustrated (Figure 1B,C)).

### 3.2. Prognostic Value of UNC5A Expression in Pan-Cancer

**Figure 2:**
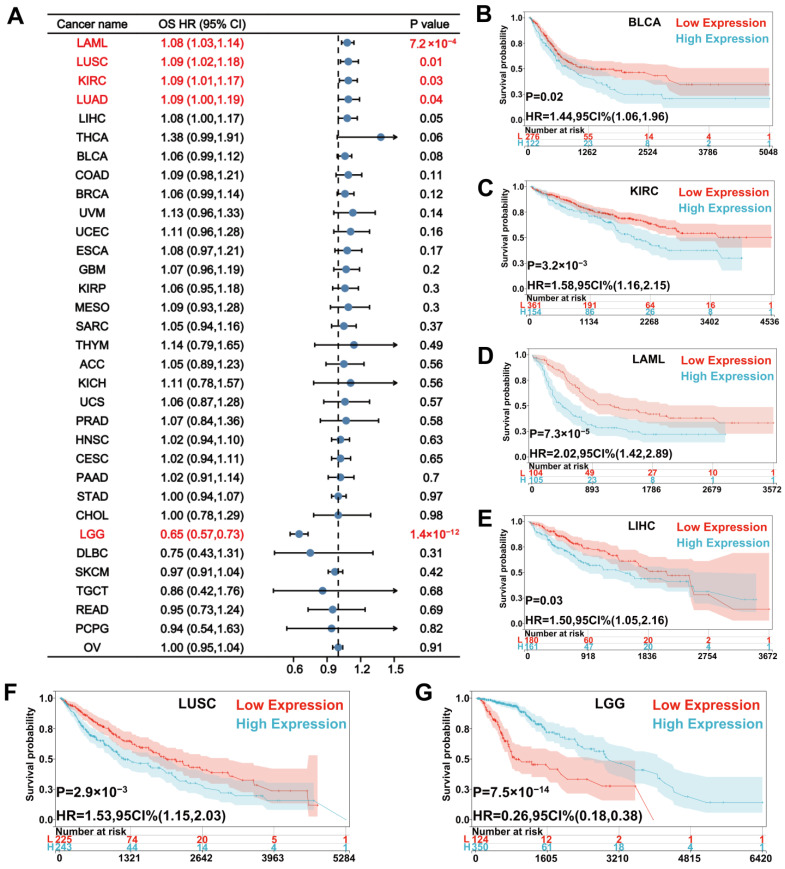
Relationship between UNC5A expression and patients’ OS in pan-cancer. (**A**) The forest plot shows the relationship of UNC5A expression with OS in pan-cancer. (**B**–**G**) Kaplan-Meier analysis shows the association between UNC5A expression and OS in pan-cancer.

For OS, Cox regression showed that a high UNC5A level was a risk factor in acute myeloid leukemia (LAML) (HR = 1.08, *p* = 7.2 × 10^−4^), LUSC (HR = 1.09, *p* = 0.01), KIRC (HR = 1.09, *p* = 0.03), and LUAD (HR = 1.09, *p* = 0.04), while it served as a protective factor in lower grade brain glioma (LGG, HR = 0.65, *p* = 1.4 × 10^−12^) (Figure 2A). Kaplan-Meier curves exhibited that patients with high UNC5A expression had worse OS than those with low UNC5A levels in bladder urothelial carcinoma (BLCA), KIRC, LAML, LUSC, and LIHC (Figure 2B–F). On the contrary, patients with low UNC5A expression had poor OS in LGG (Figure 2G).

**Figure 3:**
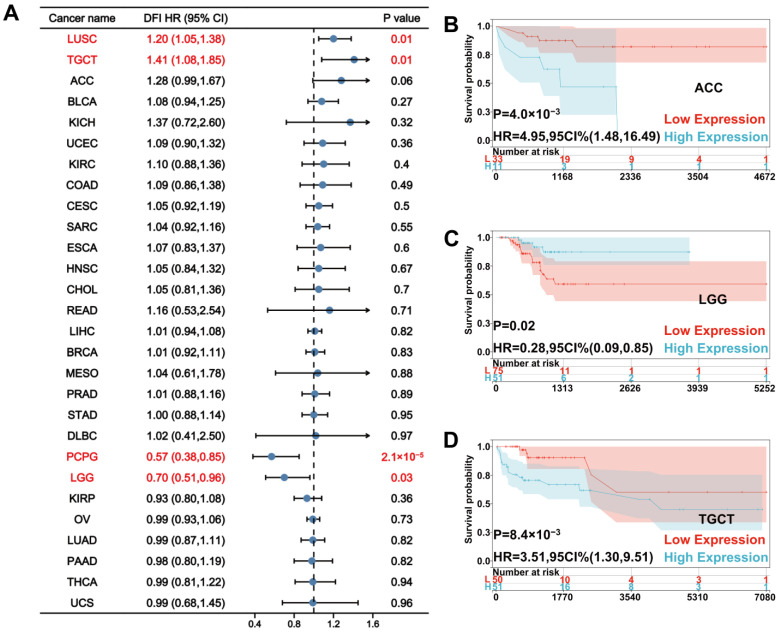
Relationship between UNC5A expression and patients’ DFI in pan-cancer. (**A**) The forest plot shows the relationship between UNC5A expression and DFI in pan-cancer. (**B**–**D**) Kaplan-Meier analysis shows the association between UNC5A expression and DFI in pan-cancer.

For DFI, Cox regression showed that a high UNC5A level was a risk factor for LUSC (HR = 1.20, *p* = 0.01) and testicular germ cell tumors (TGCT, HR = 1.41, *p* = 0.01) but served as a protective factor for pheochromocytoma and paraganglioma (PCPG, HR = 0.57, *p* = 2.1 × 10^−5^), and LGG (HR = 0.70, *p* = 0.03), as shown in Figure 3A. Kaplan-Meier curves showed that patients with high UNC5A expression had worse DFI than those with low UNC5A levels in adrenocortical carcinoma (ACC) and TGCT (Figure 3B,D). On the contrary, patients with low UNC5A expression had poor DFI and LGG (Figure 3C).

**Figure 4:**
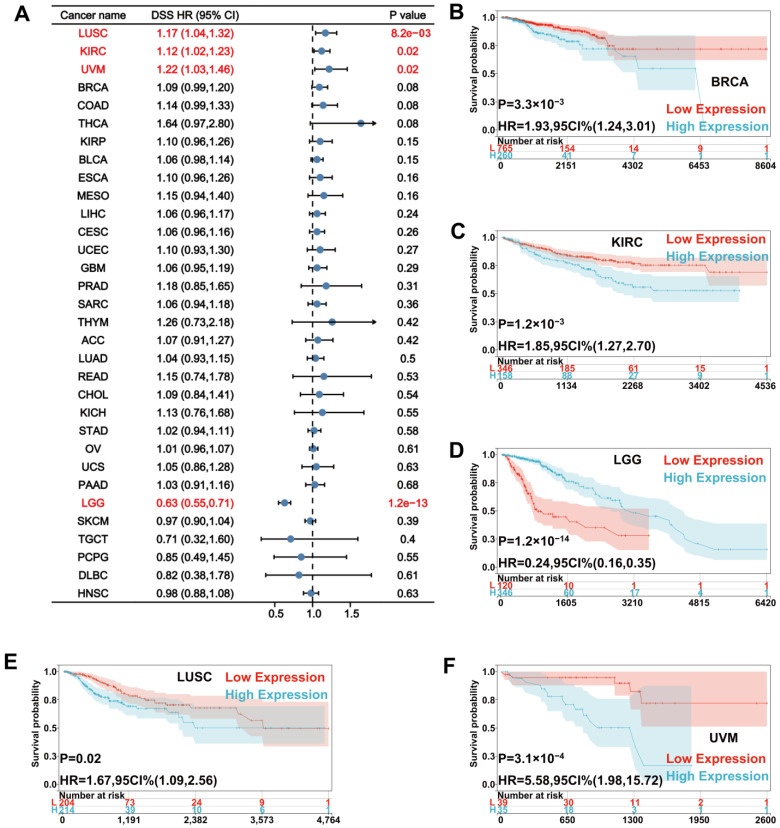
Relationship between UNC5A expression and patients’ DSS in pan-cancer. (**A**) The forest plot shows the relationship between UNC5A expression and DSS in pan-cancer. (**B**–**F**) Kaplan-Meier analysis shows the association between UNC5A expression and DSS in pan-cancer.

For DSS, Cox regression showed that a high UNC5A level was a risk factor for LUSC (HR = 1.17, *p* = 8.2 × 10^−3^), KIRC (HR = 1.12, *p* = 0.02), and uveal melanoma (UVM, HR = 1.22, *p* = 0.02) but a protective factor for LGG (HR = 0.63, *p* = 1.2 × 10^−13^) (Figure 4A). Moreover, Kaplan-Meier curves demonstrated that patients with high UNC5A expression had a worse DSS in BRCA, KIRC, LUSC, and UVM (Figure 4B,C,E,F), whereas those with low UNC5A levels had poor DSS in and LGG (Figure 4D).

**Figure 5:**
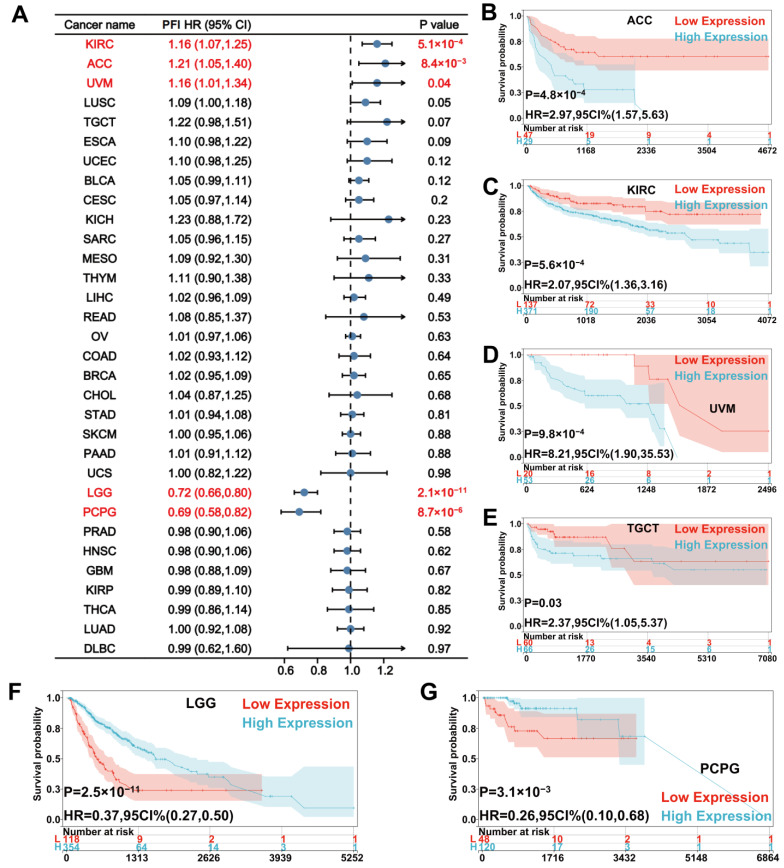
Relationship between UNC5A expression and patients’ PFI in pan-cancer. (**A**) The forest plot shows the relationship of UNC5A expression with PFI in pan-cancer. (**B**–**G**) Kaplan-Meier analysis shows the association between UNC5A expression and PFI in pan-cancer.

For PFI, Cox regression showed that a high UNC5A level was a risk factor for KIRC (HR = 1.16, *p* = 5.1 × 10^−4^), ACC (HR = 1.21, *p* = 8.4 × 10^−3^), and UVM (HR = 1.16, *p* = 0.04) but a protective factor for LGG (HR = 0.72, *p* = 2.1 × 10^−11^), and PCPG (HR = 0.69, *p* = 8.7 × 10^−6^) (Figure 5A). Kaplan-Meier curves showed that patients with high UNC5A expression had worse PFI in ACC, KIRC, TGCT, and UVM (Figure 5B–E), whereas those with low UNC5A expression had poor PFI in LGG, and PCPG (Figure 5F,G).

Subsequently, time-dependent ROC curves were plotted to examine the prognostic value of UNC5A expression in the prediction of 1-, 3-, and 5-year survival in terms of DSS, OS, and PFI in pan-cancer. UNC5A is a candidate prognostic biomarker for multiple cancer types. For example, the AUC values for predicting the corresponding 1-, 3-, and 5-year OS, DSS, and PFI of patients with KICH were (0.831, 0.603, and 0.598), (0.831, 0.653, and 0.632), and (0.665, 0.615, and 0.600), respectively. The two cancer types (KICH and UVM) with the highest AUC values are shown in Figure S1. Therefore, UNC5A may serve as an effective pan-cancer biomarker.

### 3.3. Genetic Alteration Analysis

**Figure 6:**
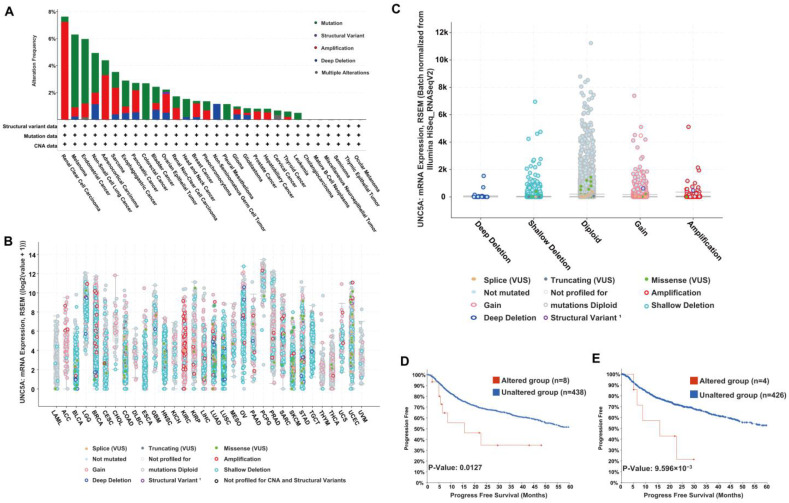
Mutational features of UNC5A in pan-cancer. (**A**) The alteration frequency by mutation type in UNC5A in pan-cancer. (**B**) The major types of UNC5A gene alterations in various cancers. (**C**) The analysis of UNC5A copy number alteration frequencies. (**D**,**E**) The relationship between PFS and UNC5A mutational status in HNSC and PRAD, respectively.

The genetic alterations in UNC5A were analyzed on cBioPortal. As shown in Figure S2, the results exhibited that UNC5A altered 285 samples (2.6%) out of 10,967 samples (data from PanCancer Atlas and TCGA). In addition, the alteration frequency of UNC5A varied in pan-cancer (Figure 6A). For instance, UNC5A had a relatively high alteration frequency in KIRC (nearly 8%), while no related alterations were found in CHOL, mature B-cell neoplasms, miscellaneous neuroepithelial tumors, seminoma, thymic epithelial tumor, and ocular melanoma. Further, the highest alteration frequency in pan-cancer was that of copy-number variations (CNV, including amplification and deep deletions) (Figure 6A), and thus, we assessed the correlation between the putative CNV in UNC5A and the corresponding gene expression in pan-cancer. The results exhibited that mutation of the UNC5A gene was intimately correlated with UNC5A transcriptional expression in pan-cancer and gene gain was the most frequency CNV of UNC5A. (Figure 6B,C). Finally, we evaluated the PFS of patients with or without UNC5A alterations across cancer types, and the results exhibited that patients with UNC5A alterations had a poor PFS in HNSC (*p* = 0.0127) and PRAD (*p* = 9.596 × 10^−3^) (Figure 6D,E).

### 3.4. PPI Networks and Functional Annotations

**Figure 7:**
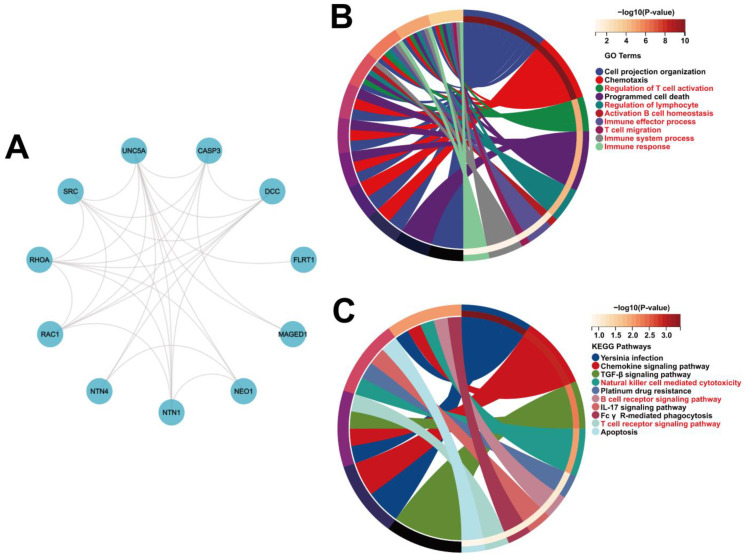
Enrichment analyses for UNC5A and the co-expression genes. (**A**) Protein-protein interaction network of UNC5A and the co-expression genes (Top 10). (**B**,**C**) GO and KEGG analyses for UNC5A and the co-expression genes.

There was a strong interaction between UNC5A and the co-expressed genes (including CASP3, DCC, FLRT1, MAGED1, NEO1, NTN1, NTN4, RAC1, RHOA, and SRC) (Figure 7A). Functional annotation was assessed. GO analysis demonstrated that UNC5A and the co-expressed genes were implicated in the “Regulation of lymphocytes”, “Regulation of T cell activation”, “Immune effector process”, “Activation of B cell homeostasis”, “Immune system process”, “T cell migration”, and “Immune response”, while KEGG analysis showed their participation in “T cell receptor signaling pathway”, “Natural killer cell-mediated cytotoxicity”, and “B cell receptor signaling pathway” (Figure 7B,C). The above results illustrated that UNC5A may participate in and regulate immunity in cancer.

### 3.5. UNC5A Expression Is Related to MSI, Neoantigen, and TMB in Pan-Cancer

**Figure 8:**
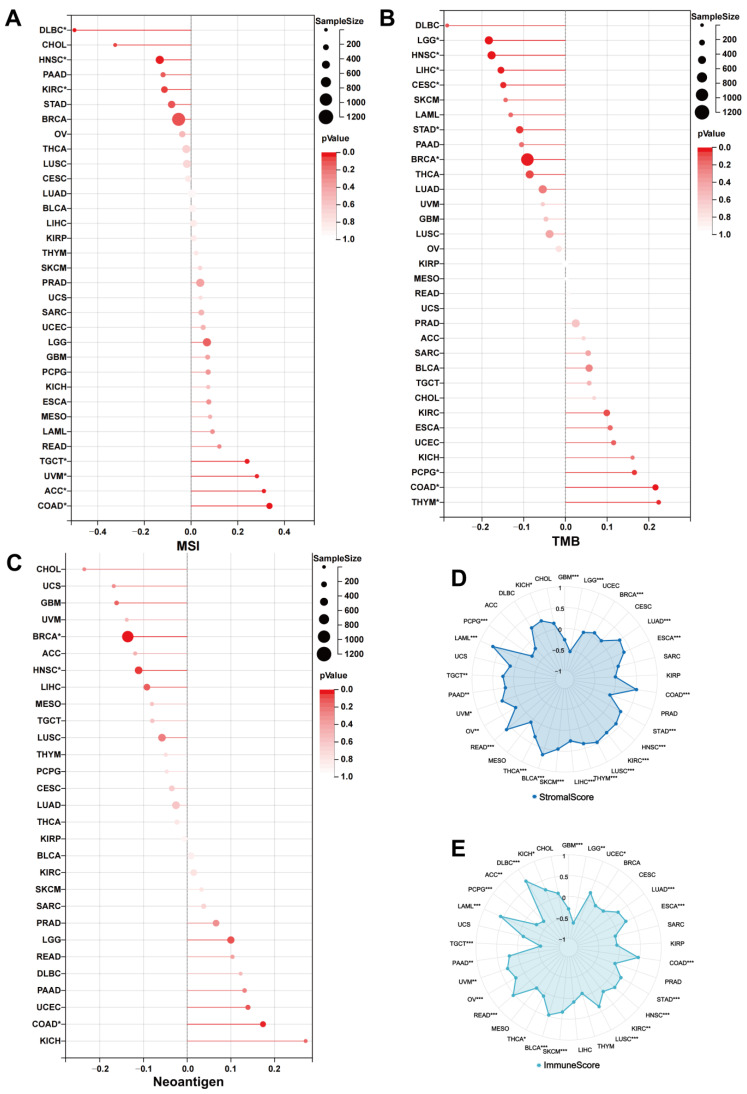
The relationship between UNC5A expression and TMB, MSI, neoantigen, and TME in pan-cancer. (**A**) The association between UNC5A expression and MSI across cancer types. (**B**) The association between UNC5A expression and TMB in pan-cancer. (**C**) The relationship between UNC5A expression and neoantigen across cancer types. (**D**,**E**) The relationship between UNC5A expression and StromalScore and ImmuneScore in pan-cancer. * *p* < 0.05, ** *p* < 0.01, *** *p* < 0.001.

Neoantigen, MSI, and TMB are strongly linked to tumorigenesis and progression and can predict tumor immunotherapeutic efficacy [28]; therefore, we studied the relationships between UNC5A expression and TMB, neoantigen, and MSI. UNC5A expression correlated positively with TMB in various cancer types such as COAD, thymoma (THYM), and PCPG, while negatively with LGG, BRCA, STAD, HNSC, cervical squamous cell carcinoma and endocervical adenocarcinoma (CESC), and LIHC (Figure 8B). UNC5A expression correlated positively with MSI in COAD, TGCT, UVM, and ACC and negatively with HNSC, KIRC, and diffuse large B-cell lymphoma (DLBC) (Figure 8A). The UNC5A expression correlated positively with neoantigen in COAD, and negatively with BRCA and HNSC (Figure 8C). These findings proved that UNC5A expression participated in tumor progression and initiation, thus serving as a potential independent biomarker for tumor immunotherapy efficacy.

### 3.6. UNC5A Expression Is Closely Related to TME in Pan-Cancer

Immune and stromal cells are crucial in TME. SangerBox was utilized to assess the association of UNC5A levels with StromalScore and ImmuneScore in pan-cancer. UNC5A expression correlated positively to both ImmuneScore and StromalScore in HNSC, rectum adenocarcinoma (READ), KIRC, THCA, UVM, esophageal carcinoma (ESCA), LAML, BLCA, COAD, LUAD, OV, STAD, prostate adenocarcinoma (PAAD), LUSC, and skin cutaneous melanoma (SKCM), while negatively in GBM, LGG, and PCPG (Figure 8D,E). Moreover, the expression of UNC5A was positively or negatively related to ImmuneScore or StromalScore in DLBC, ACC, UCEC, TGCT, LIHC, BRCA, and THYM.

### 3.7. Immune Cell Infiltration Based on UNC5A Expression in Pan-Cancer

**Figure 9:**
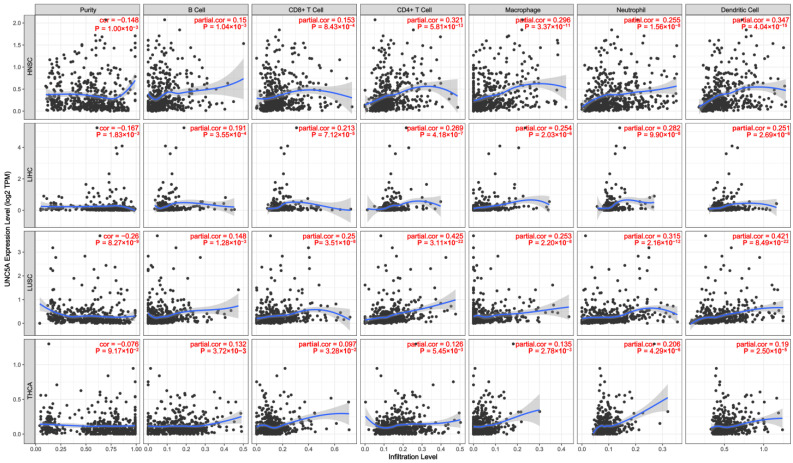
Correlation analysis between UNC5A expression and immune infiltration levels of B cells, CD8+ T cells, CD4+ T cells, macrophages, neutrophils, and dendritic cells in pan-cancer.

The immune infiltration level of UNC5A in pan-cancer was examined utilizing the TIMER database. UNC5A level was significantly related to the infiltration of B cells in 10 diverse cancer types, CD4+ T cells in 21 various cancer types, CD8+ T cells in 9 cancers, neutrophils in 18 cancers, macrophages in 16 cancers, and dendritic cells in 18 cancers. Notably, UNC5A was related to all the above six immune cell types in HNSC, LIHC, LUSC, and THCA (Figure 9). Detailed information on immune infiltration levels by UNC5A expression in other specific cancer types is presented in Figure S3. The above findings proved that UNC5A was linked to immune infiltration in pan-cancer.

### 3.8. UNC5A Expression Is Significantly Related to Immunomodulators and ICP Genes in Pan-Cancer

**Figure 10:**
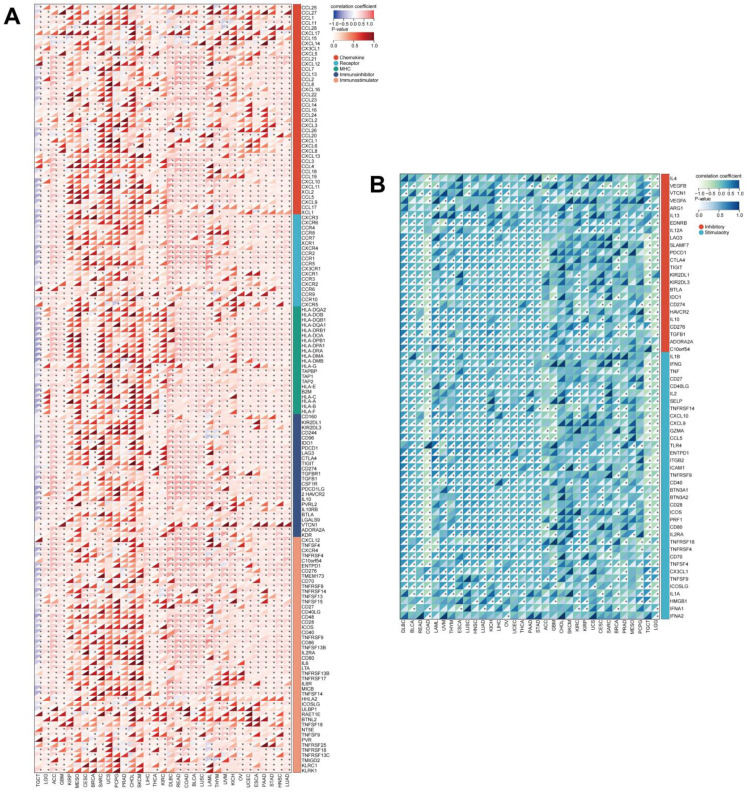
Correlations between UNC5A expression and tumor immunity. (**A**) The relationships between UNC5A expression and various immunomodulators in pan-cancer. (**B**) The relationships between UNC5A expression and various ICP genes in pan-cancer. * p < 0.05.

Next, we evaluated the relationships between UNC5A expression and immunomodulators (including chemokine, receptor, MHC, immunoinhibitor and immunostimulators) and various ICP genes across cancers. The results exhibited that UNC5A expression was substantially associated with the majority of immunomodulators in READ, COAD, BLCA, HNSC, and LUSC etc., while it was negatively related to immunomodulators in TGCT (Figure 10A). Further, the relationship between UNC5A expression and various ICP genes, with a critical role in immunotherapy and immune cell infiltration, was assessed [29]. There was a strong association between UNC5A expression and various ICP genes in pan-cancer; for example, UNC5A expression was related to 54 out of 60 ICP genes in LGG (Figure 10B). These findings illustrated that UNC5A may serve as an immunotherapy target in cancer.

### 3.9. UNC5A Expression Is Associated with DNA Methylation in Pan-cancer

**Figure 11:**
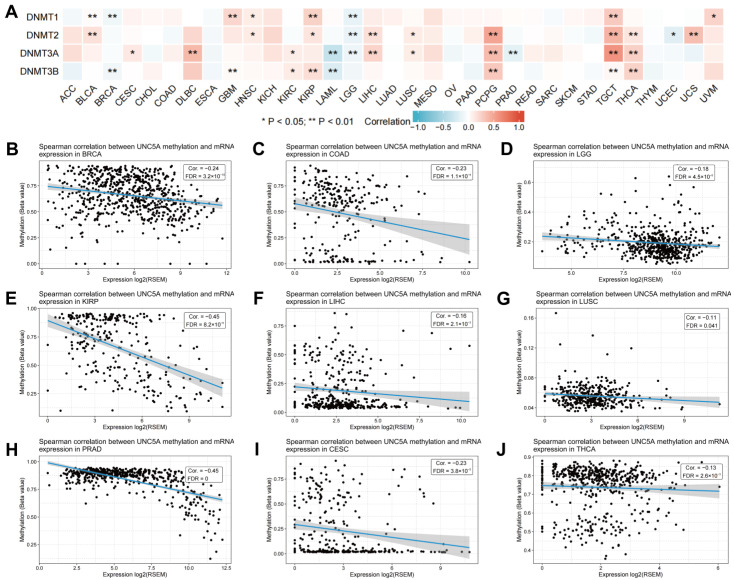
The relationship of UNC5A expression and DNA methylation status across cancer types. (**A**) Expression of UNC5A correlates with four DNA methyltransferases (DNMT1, DNMT2, DNMT3A, and DNMT3B). (**B**–**J**) Spearman correlation between UNC5A methylation and mRNA expression in BRCA, COAD, BRCA, COAD, LGG, KIRP, LIHC, LUSC, PRAD, CESC, and THCA, respectively. * p < 0.05, ** p < 0.01.

DNA methylation is crucial in tumor onset progression, as confirmed in numerous studies [30,31]. Thus, assessing the association of UNC5A level with the DNA methylation status in cancers is of great significance. First, we evaluated the association of the four DNA methyltransferases with UNC5A expression in pan-cancer and observed that UNC5A was associated with some or all four DNA methytransferases in BLCA, BRCA, CESC, DLBC, GBM, HNSC, KIRC, KIRP, LAML, LGG, LIHC, LUSC, PCPG, PRAD, TGCT, THCA, UCEC, Uterine Carcinosarcoma (UCS), and UVM (Figure 11A). Subsequently, the GSCA online website was used to elucidate the association of UNC5A expression in pan-cancer with UNC5A DNA methylation levels. The results exhibited that UNC5A expression correlated negatively with UNC5A DNA methylation levels in BRCA, COAD, LGG, KIRP, LIHC, LUSC, PRAD, CESC, and THCA (Figure 11B–J). The detailed information on UNC5A DNA methylation levels by UNC5A expression in other specific cancer types is presented in Figure S4. The above data illustrated that UNC5A may affect tumorigenesis and progression through DNA methylation.

### 3.10. Association of UNC5A Level with Drug Sensitivity

**Figure 12:**
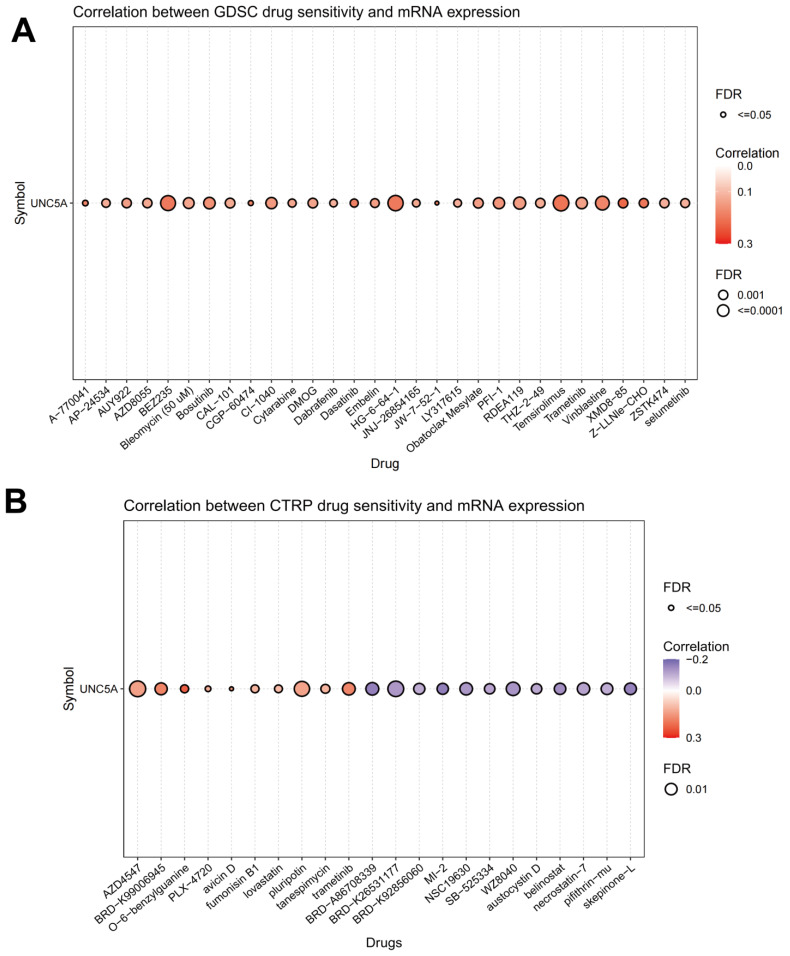
The relationship between UNC5A expression and drug sensitivity basis CTRP and GDSC databases. (**A**) UNC5A expression was positively related to various targeted or chemotherapeutic agents in GDSC database. (**B**) UNC5A expression was related to various targeted or chemotherapeutic agents in CTRP database.

The relationship between UNC5A level and drug sensitivity was investigated based on CTRP and GDSC databases, suggesting a significant association between UNC5A level and sensitivity to multiple drugs. A high level of UNC5A expression exhibited low drug sensitivity for various targeted or chemotherapeutic agents such as Bosutinib, Cytarabine, Dabrafenib, Pluripotin, and Trametinib. Conversely, a high level of UNC5A expression exhibited high drug sensitivity for Austocystin D, Belinostat, and Pifithrin-mu (Figure 12A,B). These findings may guide drug selection for some specific cancer types in clinical practice.

## 4. Discussion

Owing to the rapid development in bioinformatics, researchers can now use various analytical software and online databases (such as the R software and TCGA database) to explore potential biomarkers, signaling pathways, and therapeutic targets, and assess the relationship between specific genes with drug responses in cancer. Although several studies have reported the role of UNC5A in breast cancer, colorectal cancer, non-small cell lung cancer, bladder cancer, and gliomas, its significance in other human cancers remains unclear. Herein, the R software and multiple databases (including TCGA, GTEx, TIMER, GSCA, GEPIA, and cBioPortal) were used to examine UNC5A’s role in pan-cancer. Herein, UNC5A was highly expressed across various human cancers tissues and cells and was linked to the clinical outcomes of patients and may serve as a potential biomarker in pan-cancer. The mutational landscape of UNC5A exhibited that patients with UNC5A mutations had poor PFS in HNSC and PRAD. UNC5A expression was associated with TMB, MSI, neoantigen, TME, immune infiltration level, immunomodulator, ICP genes, DNA methylation, and drug responses.

High UNC5A expression was found in 14 human cancers. Notably, previous studies have shown that UNC5A is significantly highly expressed in adjacent non-cancerous bladder tissues [15]; however, in our study, a high expression of UNC5A was also found in normal bladder tissues but the difference was statistically insignificant, which may be attributed to the difference in the number of samples. UNC5A was related to DSS, OS, DFI, and PFI in different cancer types, and could predict the 1-, 3-, and 5-year DSS, OS, and PFI in KICH and UVM. Undoubtedly, gene mutations and epigenetic modifications play important roles in tumorigenesis and progression [32,33,34,35]. Gene alterations in UNC5A occurred in the majority of cancer types, especially KIRC (alteration frequency was nearly 8%), and UNC5A alterations were related to the PFS of patients with HNSC and PRAD. One of the ligands of UNC5A, netrin-1 is known to be overactivated in cancers, especially in inflammation-driven tumors. For instance, Paradisi et al. demonstrated that inflammation-driven netrin-1 up-regulation is causal for colorectal cancer development [36]. In addition, a recent study found that netrin-1 regulates inflammation and leukocyte infiltration, suggesting roles for netrin-1 in the immune response [37,38,39,40]. Due to the close relationship between UNC5A and netrin-1, we explored the relationship between UNC5A and tumor immunity in cancers. First, we analyzed the functional annotations for UNC5A and the co-expressed genes. Surprisingly, they primarily participated in immune regulation. Subsequently, the association of UNC5A level with TMB, neoantigen, and MSI was evaluated, and our findings showed the UNC5A expression was related to TMB, MSI, and neoantigenin pan-cancer. These findings illustrated that UNC5A may play a crucial role in tumor immunity. In addition, it is an independent biomarker for predicting the efficacy of tumor immunotherapy.

TME is a complex system, where the TIME is an essential constituent intimately correlated with tumor immunity and is implicated in tumor progression [41,42,43]. TIICs can predict the prognosis and efficacy of immunotherapy in cancer [44,45]. Herein, UNC5A expression was found to be significantly related to CD8+ T cells in 9 cancers, B cells in 10 diverse cancer types, CD4+ T cells in 21 cancers, macrophages in 16 cancers, neutrophils in 18 cancer types, and dendritic cells in 18 cancer types. Notably, UNC5A levels were related to all selected immune cell types in HNSC, LIHC, LUSC, and THCA. The relationship between UNC5A expression with the majority of immunomodulators and ICP genes was significant, especially in LGG, suggesting that UNC5A may be a potential immunotherapeutic target and might serve vital functions in tumor immunotherapy responses and outcomes.

DNA methylation is a crucial epigenetic change implicated in tumorigenesis and development [46,47]. Generally, human tumors exhibit genome-wide hypomethylation, causing activation and induction of tumorigenesis due to the methylation of proto-oncogene promoters [48,49]. Herein, we first assessed the relationship between the four DNA methyltransferases and UNC5A expression and found significant pan-cancer associations. UNC5A expression correlated negatively with UNC5A DNA methylation levels in various cancer types, hinting at the potential tumorigenesis mechanism of UNC5A action in pan-cancer. The choice of anti-tumor drugs is crucial for precise and individualized treatment. Finally, we assessed the relationship between UNC5A expression and drug sensitivity. UNC5A expression correlated significantly with sensitivity to different small-molecule drugs. Therefore, choosing appropriate therapeutic drugs according to the levels of UNC5A across tumors may bear clinical significance.

Although we comprehensively analyzed UNC5A expression in pan-cancer, some limitations warrant further consideration. First, the data utilized in this study were derived from open sources, and in vitro/in vivo experiments, and other public databases are needed to validate our conclusion. Second, UNC5A is highly expressed and related to clinical outcomes in pan-cancer; however, its potential mechanism of action has not yet been elucidated. Thus, further investigations are needed to evaluate the potential action mechanism of UNC5A in the diagnosis and patient prognosis of cancers. Finally, in this study we found that there is a tight relationship between UNC5A and tumor immunity. However, how UNC5A’s relation to tumor immunity has not been elucidated, which should be further explored in future studies.

## 5. Conclusions

**Figure 13:**
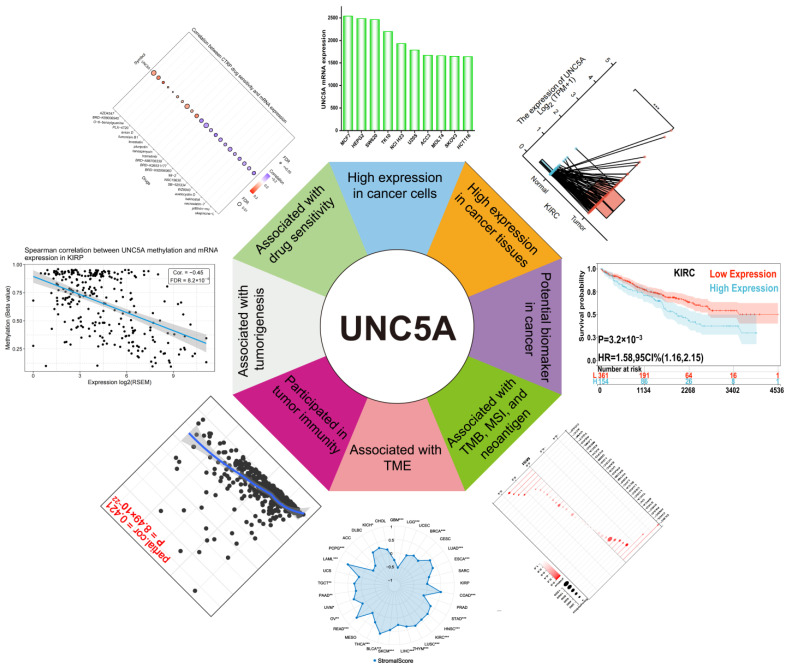
Summary of main findings in this work. UNC5A is highly expressed in cancer cells and tissues. High UNC5A expression is associated with poor clinical outcomes. Meanwhile, UNC5A is related to TMB, MSI, neoantigen, TME, and tumor immunity. In addition, UNC5A may participate in tumorigenesis though DNA methylation. Finally, UNC5A is associated with drug sensitivity. * *p* < 0.05, ** *p* < 0.01, *** *p* < 0.001.

In summary, we present the first comprehensive role of UNC5A in pan-cancer. As shown in Figure 13, our findings demonstrated a high expression of UNC5A across pan-cancers and its relationship with several clinical outcomes. UNC5A expression was significantly associated with MSI, TMB, neoantigen, TME, and tumor immunity in pan-cancer. Finally, the UNC5A may promote tumorigenesis and progression through DNA methylation and its expression can guide drug selection for some specific cancer types in clinical practice.

## Data Availability

The data used to support the findings of this study are included in the article.

## Supplementary Materials

The following supporting information can be downloaded at: https://www.mdpi.com/article/10.3390/biom12121826/s1, Figure S1. Prognostic significance of UNC5A in KICH and UVM. (**A**–**C**) the prognostic value of UNC5A for predicting 1-, 3-, and 5-year of OS, DSS, and PFI in KICH, respectively. (**D**–**F**) the prognostic value of UNC5A for predicting 1-, 3-, and 5-year of OS, DSS, and PFI in UVM, respectively. Figure S2. Summary of alterations in UNC5A expression in different tumors. Figure S3. Relationship between UNC5A expression and immune infiltration level in BLCA, COAD, KIRC, LGG, and LUAD, respectively. Figure S4. (**A**–**M**) Spearman correlation between UNC5A methylation and mRNA expression in BLCA, DLBC, GBM, LAML, PAAD, PCPG, READ, STAD, TGCT, THYM, UCS, and UVM, respectively.
