# Supplementary material for: A Systematic Analysis of the Role of Unc-5 Netrin Receptor A (UNC5A) in Human Cancers
Source: Biomolecules. 2022 Dec 6;12(12):1826. doi: 10.3390/biom12121826 (PMC9775303; doi:10.3390/biom12121826)
Supplement: Supplementary file 1 [file biomolecules-12-01826-s001.zip › supplementary materials.pdf]

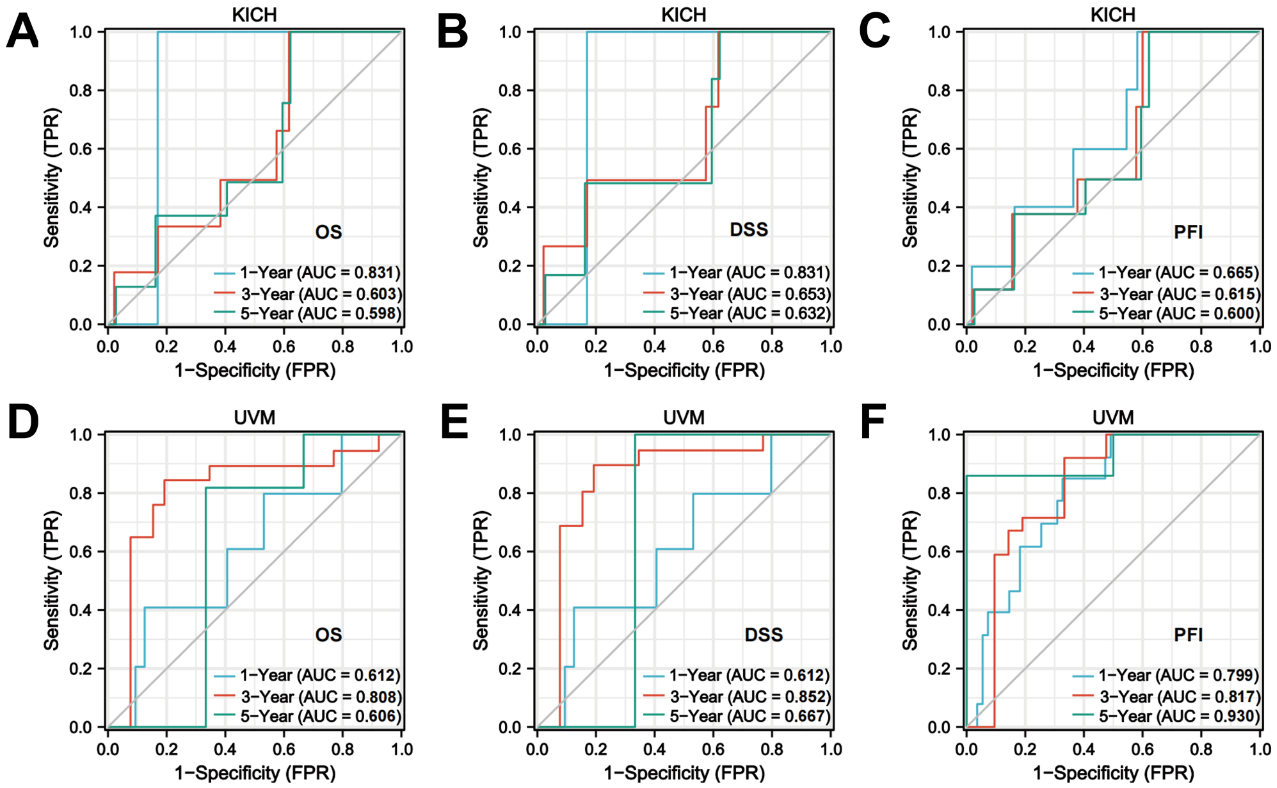

Figure S1. Prognostic significance of UNC5A in KICH and UVM. (A) - (C) the prognostic value of UNC5A for predicting 1-, 3-, and 5-year of OS, DSS, and PFI in KICH, respectively. (D) - (F) the prognostic value of UNC5A for predicting 1-, 3-, and 5-year of OS, DSS, and PFI in UVM, respectively.

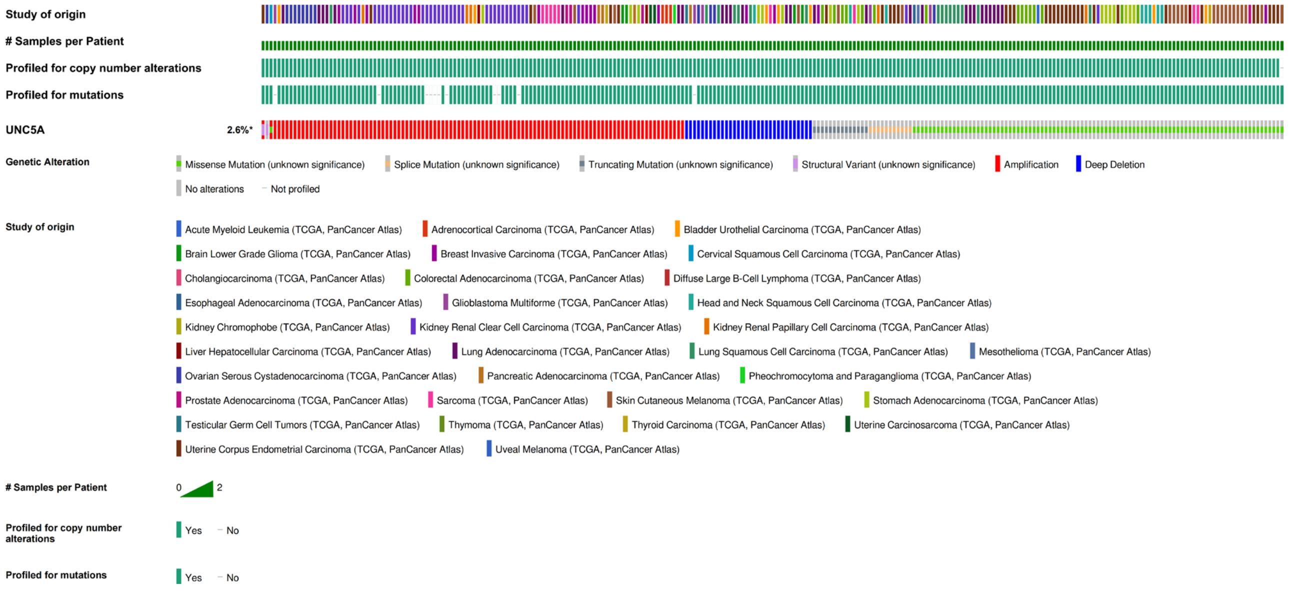

Figure S2. Summary of alterations in UNC5A expression in different tumors

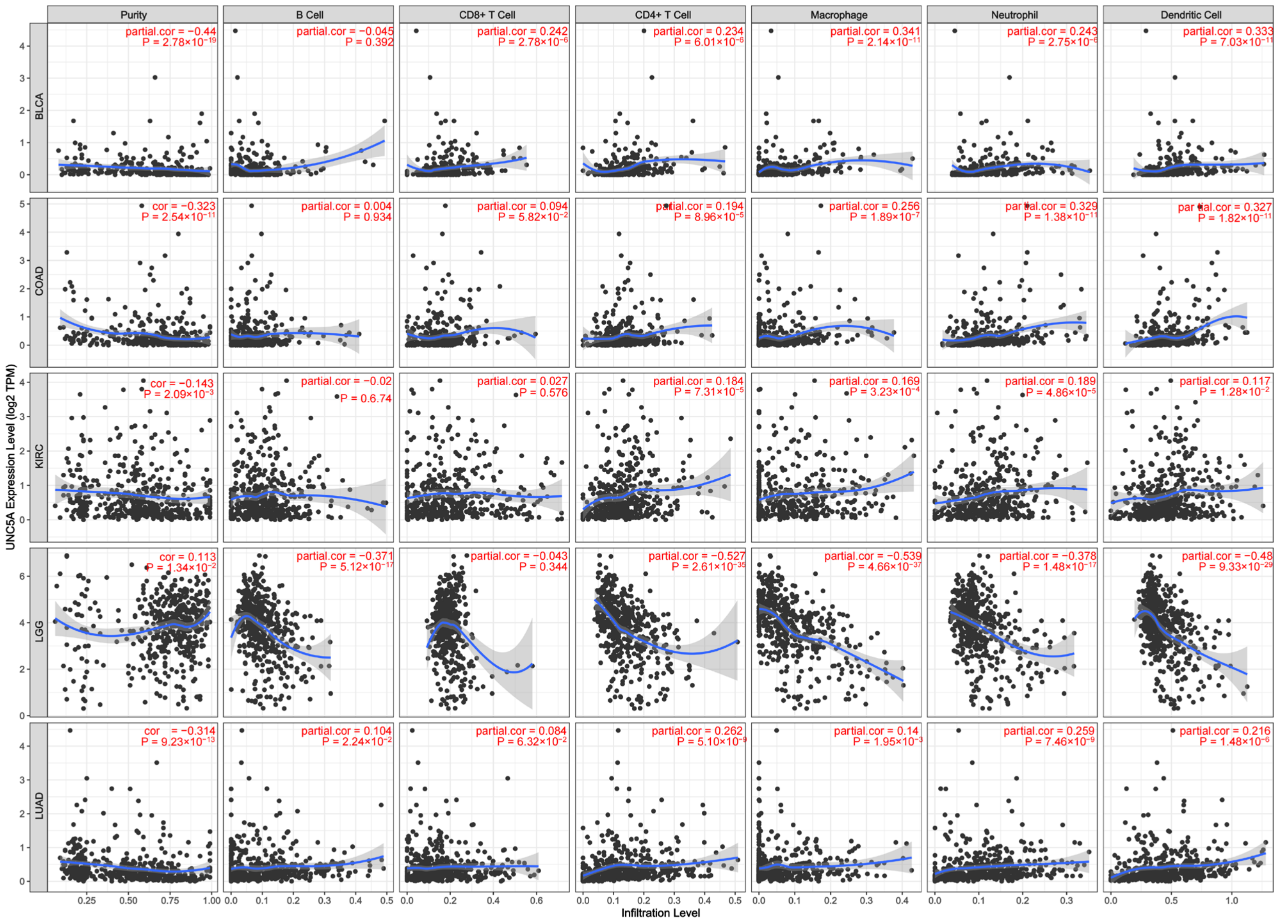

Figure S3. Relationship between UNC5A expression and immune infiltration level in BLCA, COAD, KIRC, LGG, and LUAD, respectively.

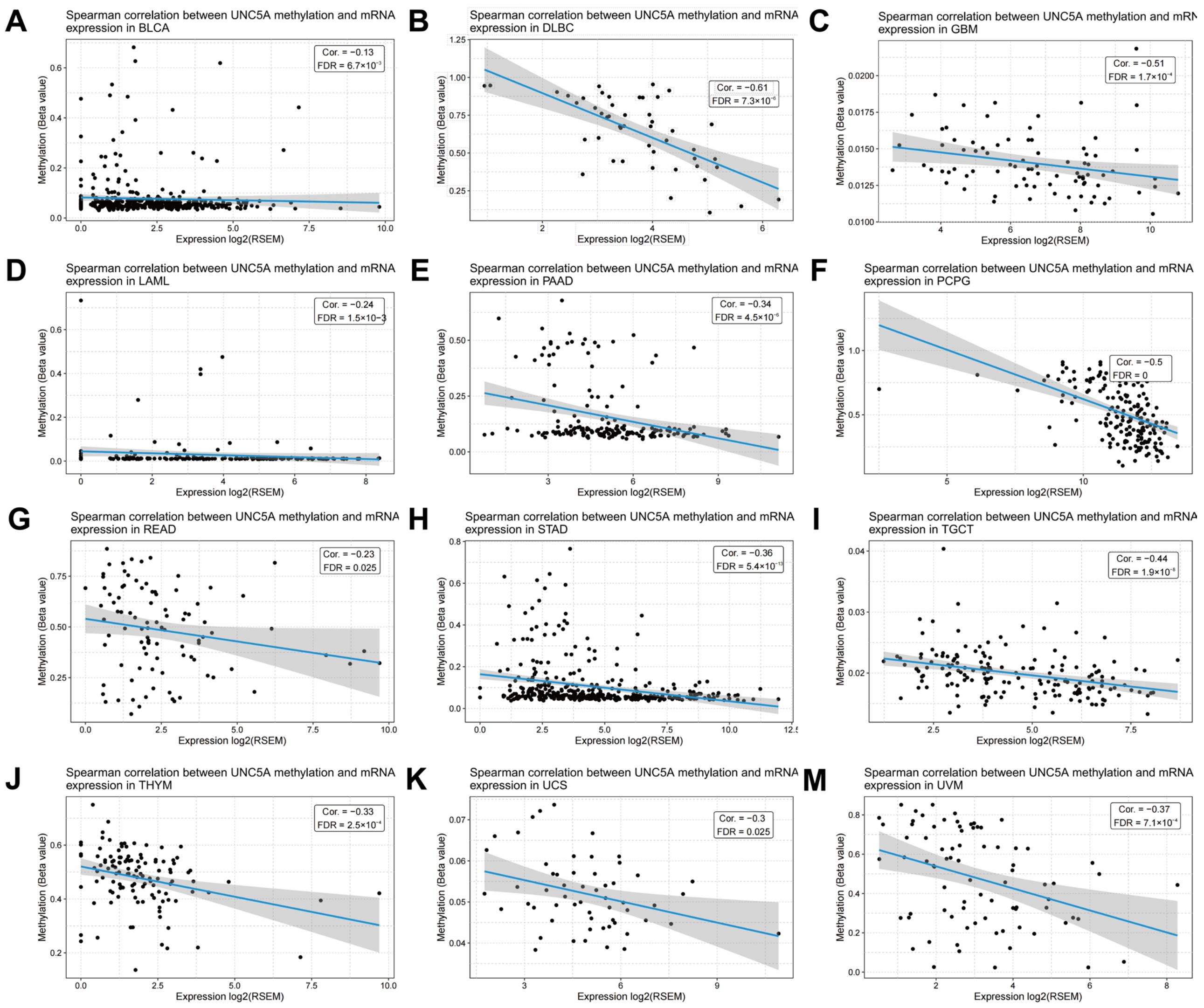

Figure S4. (A)- (M) Spearman correlation between UNC5A methylation and mRNA expression in BLCA, DLBC, GBM, LAML, PAAD, PCPG, READ, STAD, TGCT, THYM, UCS, and UVM, respectively.
